# Assessment of Cervical Abrasion, Dentin Hypersensitivity, and Its Treatment Needs Using the Cervical Abrasion Index of Treatment Needs Probe

**DOI:** 10.7759/cureus.33471

**Published:** 2023-01-06

**Authors:** Abdul Salam T A, Sheeja S Varghese, Rekha P Shenoy

**Affiliations:** 1 Public Health Dentistry, Saveetha Dental College and Hospitals, Saveetha University, Chennai, IND; 2 Periodontology, Saveetha Dental College and Hospitals, Saveetha University, Chennai, IND; 3 Public Health Dentistry, Yenepoya Dental College, Mangalore, IND

**Keywords:** cervical abrasion, treatment needs, caitn probe, buccolingual measurements, hypersensitivity

## Abstract

Objective

This study was intended to compare the mean buccolingual measurement of abraded teeth with/without sensitivity. The hypothesis was that the suggested treatment approaches would be in agreement with that of the treatment needs (TN) elicited using the Cervical Abrasion Index of Treatment Needs (CAITN) probe and aid in the development of a prospective CAITN index for clinical/community studies of effective therapeutic measures.

Materials and methods

A cross-sectional study was carried out on 30 individuals with a mean age of 48.4±12.54 years, comprising 840 teeth with/without cervical abrasion. The buccolingual measurement of each tooth was recorded using the CAITN probe. The response to air-blast was assessed by a short blast of one-second duration at a distance of 1 cm for each tooth. An endodontist was also asked to indicate the treatment methods used by him for the treatment of abrasions. His opinions regarding the TN were later compared with the data collected by an investigator.

Results

The mean buccolingual measurements of all the teeth were compared with the dentinal sensitivity using the independent t-test and were statistically highly significant. Furthermore, one-way analysis of variance disclosed that there was a statistically highly significant difference found for all the TN (p<0.01) such as none, preventive, basic, and advanced restorative TN followed by Tukey honestly significant difference post hoc tests for multiple pair-wise comparisons. The running receiver operator characteristic curve discloses the best cut-off value of the buccolingual measurement to predict the various categories of TN of each tooth. As the area under the curve is more close to 1 (noticed in the majority of the teeth in the sample), the model predicts the TN more precisely based on the buccolingual measurements.

Conclusion

The present study enables a correct diagnosis of cervical abrasions and determines the various TN with the most appropriate restorative material. These baseline data help to design clinical studies that test relevant treatment and diagnostic strategies.

## Introduction

Cervical abrasion refers to the persistent, gradual loss of tooth structure unrelated to the caries process that occurs due to the mechanical wear of teeth at the cementoenamel junction [1]. Dentin hypersensitivity (DH) resulting from abrasion is characterized by a sharp, short-term pain that is induced by the simple touch of an instrument, toothbrush bristles, cold or sweet foods, beverages, or any other non-noxious environmental stimuli [2]. It is a condition in which the exposed vital dentin has an aberrant response to physical, chemical, or tactile stimuli [3]. The wide range in prevalence (1-98%) is most likely due to differences in the parameters used to identify DH, such as the data collection methods from questionnaires rather than reliable clinically based metrics and patient histories [4,5]. Such frequent occurrences should not be ignored, as they have become a public health problem. The lesions are not only an aesthetic problem but also a functional one, with the possibility of a loss of masticatory units [6].

DH is one of the most vexing dental problems, affecting people aged 20 to 50 [7,8]. The hydrodynamic theory explains the environmental, mechanical, thermal, and chemical changes that cause fluid movement within the exposed dentinal tubules, stimulating the pulpal fibers and inducing transient sharp pain. Visual or tactile examination of the teeth is essential to elicit the characteristic DH by applying a stimulus to the affected tooth with standardized air-blast stimulation [4,7,9].

The typically wedge-shaped hypersensitive cervical abrasion lesions involve a wide array of treatment options, each of which has its own limitations [10]. The diversified array of management strategies for abrasion implies that no single treatment meets all of the optimal characteristics. The line of treatment appears to be based primarily on the practitioner's judgment and personal inclinations [3,11]. Henceforth, this study was conducted to find out the diagnostic and treatment needs (TN) for DH commonly used by endodontists in a clinical setting. It is also intended to compare the mean buccolingual measurement of abraded teeth with sensitivity with that of abraded teeth without sensitivity. The hypothesis was that the suggested treatment approaches by the endodontist would be in agreement with the TN elicited based on the buccolingual measurement using the Cervical Abrasion Index of Treatment Needs (CAITN) probe, which would then later aid in the development of prospective clinical or community studies of effective therapeutic measures.

## Materials and methods

A cross-sectional study was carried out in a dental setting (PMS College of Dental Science & Research, Thiruvananthapuram) in Kerala to determine the buccolingual measurement of the abraded teeth using the CAITN probe. Written informed consent to participate in the study was obtained from the study subjects, and a clinical examination was carried out. Ethical clearance was provided by Saveetha Dental College with IRB number SDC/PhD18/33. Subjects who had a medical condition or were using a medication such as an antidepressant or analgesic that could interfere with reliable pain reporting had used a desensitizing dentifrice within the preceding four weeks and had received an anti-hypersensitivity treatment of the identified tooth within the preceding four weeks, Hypersensitive teeth that had carious lesions or irreversible pulpitis, vertical cracks or fractures in enamel, partial denture clasps on the facial surface, the full crown, or evidence of inflamed gingival tissue were excluded from the study. Patients above 20 years of age who met the eligibility criteria were enrolled in the study, thus forming a convenience sample.

The study was conducted on 30 subjects, comprising 15 females with a mean age of 49.5 +10.5 years and 15 males with a mean age of 47.3 +14.58 years, resulting in the evaluation of 840 teeth with or without cervical abrasion. The power analysis of the sample was higher than 90%, which was carried out after the calculation of the mean and standard deviation of the DH score using the software G*Power 3.1.9.2 (Heinrich Heine University Düsseldorf, Düsseldorf, Germany).

The buccolingual measurement of each tooth was performed using the CAITN probe [12,13]. The depth of the lesion was measured by placing the probe perpendicular to the long axis of the tooth at the center of the lesion. The presence of DH was evaluated with standardized air-blast stimulation of pressure 40 ± 5 pound-force per square inch (psi) using an air syringe. The response of the patient to an air blast was assessed by a short blast of one-second duration at a distance of 1 cm for each affected tooth, parallel to the occlusal surface from the vestibular aspect at the occluso-gingival height of the lesion. The principal investigator protected the adjacent teeth with fingers or cotton rolls. The subjective patient response to the presence of sensitivity was recorded.

As part of the study, an endodontist was also asked to indicate which treatment methods he used for the treatment of cervical abrasion. The endodontist indicated his opinions regarding the TN, which were later compared with the data collected by the investigator.

Clinical assessment

Clinical assessments and evaluations of all the teeth with cervical abrasion identified visually or tactilely were conducted by the principal investigator. We performed an initial calibration to ensure standardization at baseline and at different times of the same day to measure the buccolingual measurement using the CAITN probe and to assess the DH intensity using an air blast. The calibration was carried out in a dental setting using a dental mannequin (Chesa, Bengaluru, Karnataka), an air-water syringe, and a CAITN probe. The calibration process, such as training and adjustments, was performed on 10 patients with cervical abrasion, and the presence or absence of the condition was recorded. The intra-examiner Kappa value was calculated using the baseline values for buccolingual measurement and DH, followed by reexamining the patients seven days later. The Kappa value was determined to be 0.85.

Statistical analysis

The obtained results have been statistically analyzed using IBM SPSS Statistics for Windows, Version 26 (Released 2019; IBM Corp., Armonk, New York, United States). The results were analyzed using the independent sample t-test to compare the mean buccolingual measurement of abraded teeth with sensitivity with that of abraded teeth without sensitivity. A one-way analysis of variance (ANOVA) was carried out to compare the significant difference between the mean buccolingual measurement and the TN, followed by Tukey honestly significant difference (HSD) post hoc tests for multiple pair-wise comparisons. Furthermore, receiver operating characteristic (ROC) analysis is needed to determine the cut-off value for predicting the none, preventive TN, basic restorative, advanced restorative, and rehabilitative TN based on the buccolingual measurements. The significance level is set at p 0.05. 

## Results

The mean age of the study sample was found to be 48.4 ± 12.54 years of age. Table 1 and Table 2 show that the mean buccolingual measurements of all the teeth were compared with the DH using the independent sample t-test and were found to be statistically highly significant (p<0.01).

**Table 1:** Independent sample t-test for comparison of buccolingual measurements and dentin hypersensitivity of the maxillary arch SD: standard deviation **: statistically highly significant

Side		Presence of Sensitivity			Absence of Sensitivity			p-value
		N	Mean	SD	N	Mean	SD	
Central Incisor	Right	6	3.67	1.366	24	6.42	1.060	<0.001**
	Left	4	4.50	2.887	26	6.31	1.158	<0.001**
Lateral Incisor	Right	6	3.00	1.549	24	5.58	0.974	0.008**
	Left	8	2.75	1.581	22	5.36	1.590	<0.001**
Canine	Right	6	3.67	1.366	24	6.75	1.567	<0.001**
	Left	12	3.50	1.446	18	6.67	1.749	<0.001**
First Premolar	Right	10	2.60	2.171	20	7.00	1.376	<0.001**
	Left	16	4.00	1.461	14	8.00	1.359	<0.001**
Second Premolar	Right	14	3.43	1.453	16	7.75	1.342	<0.001**
	Left	14	4.29	1.204	16	8.50	1.033	<0.001**
First Molar	Right	14	6.57	1.742	16	11.13	0.619	<0.001**
	Left	14	5.57	2.472	16	11.63	1.025	<0.001**
Second Molar	Right	6	6	0.89	24	10.58	1.412	<0.001**
	Left	0	-	-	30	10.80	0.761	-

**Table 2:** Independent sample t-test for comparison of buccolingual measurements and dentin hypersensitivity of the mandibular arch SD: standard deviation **: statistically highly significant

Buccolingual measurements of mandibular arch								
	Side	Presence of sensitivity			Absence of sensitivity			p-value
		N	Mean	SD	N	Mean	SD	
Central Incisor	Right	4	5.50	.577	26	5.77	.430	0.272
	Left	2	5.00	.000	28	5.64	.911	0.335
Lateral Incisor	Right	8	3.75	1.753	22	5.36	1.177	0.007**
	Left	6	3.33	1.366	24	5.67	.761	0.007**
Canine	Right	8	3.50	1.604	22	6.55	1.184	<0.001**
	Left	6	4.67	.516	24	7.25	1.391	<0.001**
First Premolar	Right	10	3.80	1.687	20	6.20	1.281	<0.001**
	Left	10	4.20	1.033	20	6.80	1.436	<0.001**
Second Premolar	Right	12	3.17	1.642	18	6.67	1.283	<0.001**
	Left	14	2.86	1.512	16	5.50	1.633	<0.001**
First Molar	Right	14	5.00	2.828	16	8.88	1.204	<0.001**
	Left	12	5.17	2.290	18	9.78	.647	<0.001**
Second Molar	Right	2	3.00	.000	2	3.00	.000	<0.001**
	Left	4	3.50	.577	26	9.00	.000	<0.001**

Furthermore, the one-way ANOVA results revealed a statistically significant difference between buccolingual measurements and for all TN groups (p<0.01), including none, preventive, basic, advanced restorative, and rehabilitative TN (Table 3 and Table 4).

**Table 3:** One-way ANOVA to compare mean buccolingual measurement and treatment needs of the maxillary arch SD: Standard deviation; ANOVA: analysis of variance **: statistically highly significant

			None	Preventive	Basic Restorative	Advanced Restorative	Rehabilitative	p-value
Central Incisor	Right	N	18	2	10	-	-	<0.001**
		Mean	7	5	4	-	-	
		SD	0.00	0.00	1.155	-	-	
	Left	N	18	2	8	2	-	<0.001**
		Mean	7	7	4.75	2	-	
		SD	0.00	0.00	0.886	0.00	-	
Lateral Incisor	Right	N	20	2	8	-	-	<0.001**
		Mean	6	5	2.75	-	-	
		SD	0.00	0.00	0.886	-	-	
	Left	N	10	2	14	4	-	<0.001**
		Mean	7	5	3.57	2.5	-	
		SD	0.00	0.00	0.938	1.732	-	
Canine	Right	N	16	2	12	-	-	<0.001**
		Mean	7.75	5	4.17	-	-	
		SD	0.447	0.00	1.267	-	-	
	Left	N	10	-	16	2	2	<0.001**
		Mean	8	-	4.5	2	3	
		SD	0.00	-	0.00	1.461	0.00	
First Premolar	Right	N	10	-	16	4	-	<0.001**
		Mean	8.2	-	5	1	-	
		SD	0.422	-	1.789	0.000	-	
	Left	N	8	-	22	-	-	<0.001**
		Mean	9	-	4.73	-	-	
		SD	0.000	-	1.804	-	-	
Second Premolar	Right	N	8	2	16	4	-	<0.001**
		Mean	9	6	5	2	-	
		SD	0.00	0.00	1.713	0.00	-	
	Left	N	12	-	16	2	-	<0.001**
		Mean	9	-	5.25	2	-	
		SD	0.00	-	1.342	0.000	-	
First Molar	Right	N	14	-	14	2	-	<0.001**
		Mean	11.29	-	7.29	5	-	
		SD	0.469	-	1.978	0.00	-	
	Left	N	14	2	12	2	-	<0.001**
		Mean	12	10	5.83	3	-	
		SD	0.000	0.000	2.125	0.000	-	
Second Molar	Right	N	24	6	-	-	-	<0.001**
		Mean	10.58	6	-	-	-	
		SD	1.412	0.894	-	-	-	
	Left	N	26	-	4	-	-	<0.001**
		Mean	11	-	9.50	-	-	
		SD	0.000	-	1.732	-	-	

**Table 4:** One-way ANOVA to compare mean buccolingual measurement and treatment needs of the mandibular arch SD: Standard deviation; N: none; ANOVA: analysis of variance **: statistically highly significant

			None	Preventive	Basic Restorative	Advanced Restorative	Rehabilitative	p-value
Central Incisor	Right	N	20	2	6	2	-	-
		Mean	6	5	5	6	-	
		SD	0.000	0.000	0.000	0.000	-	
	Left	N	24	2	4	-	-	<0.001**
		Mean	6	5	3.5	-	-	
		SD	0.000	0.000	0.577	-	-	
Lateral Incisor	Right	N	16	-	14	-	-	<0.001**
		Mean	6	-	3.71	-	-	
		SD	0.000	-	1.437	-	-	
	Left	N	20	2	6	2	-	<0.001**
		Mean	6	5	3	4	-	
		SD	0.000	0.000	0.894	0.000	-	
Canine	Right	N	18	-	6	6	-	<0.001**
		Mean	7	-	4	3.67	-	
		SD	0.000	-	1.167	1.366	-	
	Left	N	18	2	6	4	-	<0.001**
		Mean	8	5	4.33	5.50	-	
		SD	0.000	0.000	0.516	0.577	-	
First Premolar	Right	N	8	-	16	6	-	<0.001**
		Mean	7.5	-	5.13	3.33	-	
		SD	0.535	-	0.957	1.862	-	
	Left	N	10	-	14	6	-	<0.001**
		Mean	8	-	5.14	4.33	-	
		SD	0.000	-	1.167	1.366	-	
Second Premolar	Right	N	12	2	10	6	-	<0.001**
		Mean	7.33	6	4.60	2	-	
		SD	0.492	0.000	1.430	0.894	-	
	Left	N	6	2	14	8	-	<0.001**
		Mean	7	6	3.86	2.50	-	
		SD	0.000	0.000	1.406	1.604	-	
First Molar	Right	N	12	2	12	4	-	<0.001**
		Mean	9.33	10	5.67	3	-	
		SD	0.492	0.000	2.146	2.309	-	
	Left	N	16	-	10	4	-	<0.001**
		Mean	10	-	6.20	4	-	
		SD	0.000	-	2.530	0.000	-	
Second Molar	Right	N	26	-	2	2	-	-
		Mean	9	-	6	3	-	
		SD	0.000	-	0.000	0.000	-	
	Left	N	26	-	2	2	-	-
		Mean	9	-	4	3	-	
		SD	0.000	-	0.000	0.000	-	

As shown in Table 5 and Table 6, Tukey HSD post hoc tests for multiple pair-wise comparisons revealed a highly significant difference between the different TN and buccolingual measurements (p<0.01).

**Table 5:** Tukey HSD post hoc tests for multiple pair-wise comparisons of the buccolingual measurements of the maxillary teeth N: None; P: preventive treatment need; B: basic restorative need; A: advanced restorative need; R: rehabilitative need; HSD: honestly significant difference *significant; **highly significant

Maxillary arch										
		N-P	N-B	P-B	N-A	B-A	P-A	N-R	B-R	A-R
Central Incisor	Right	0.001**	<0.001**	0.148	-	-	-	-	-	-
	Left	0.999	<0.001**	<0.001**	<0.001**	<0.001**	<0.001**	-	-	-
Lateral Incisor	Right	0.016*	<0.001**	<0.001**	-	-	-	-	-	-
	Left	0.034	<0.001**	0.170	<0.001**	0.170	0.016*	-	-	-
Canine	Right	0.001**	<0.001**	0.437	-	-	-	-	-	-
	Left	-	<0.001**	-	<0.001**	0.028*	-	<0.001**	0.295	0.804
First Premolar	Right	-	<0.001**	-	<0.001**	<0.001**	-	-	-	-
	Left	-	-	-	-	-	-	-	-	-
Second Premolar	Right	0.034*	<0.001**	0.737	<0.001**	0.002**	0.008**	-	-	-
	Left	-	<0.001**	-	<0.001**	0.001**		-	-	-
First Molar	Right	-	<0.001**	-	<0.001**	0.100		-	-	-
	Left	0.247	<0.001**	0.003	<0.001**	0.057	<0.001**	-	-	-
Second Molar	Right	-	-	-	-	-	-	-	-	-
	Left	-	-	-	-	-	-	-	-	-

**Table 6:** Tukey HSD post hoc tests for multiple pair-wise comparisons of the buccolingual measurements of the mandibular teeth N: None; P: preventive treatment need; B: basic restorative need; A: advanced restorative need; R: rehabilitative need; HSD: honestly significant difference *significant; **highly significant

Mandibular arch										
		N-P	N-B	P-B	N-A	B-A	P-A	N-R	B-R	A-R
Central incisor	Right	-	-	-	-	-	-	-	-	-
	Left	<0.001**	<0.001**	<0.001**	-	-	-	-	-	-
Lateral incisor	Right	-	-	-	-	-	-	-	-	-
	Left	0.010	<0.001**	<0.001**	<0.001**	0.021*	0.075	-	-	-
Canine	Right	-	<0.001**	-	<0.001**	0.845		-	-	-
	Left	<0.001**	<0.001**	0.052	<0.001**	<0.001**	0.242	-	-	-
First Premolar	Right	-	<0.001**	-	<0.001**	0.006**		-	-	-
	Left	-	<0.001**	-	<0.001**	0.240		-	-	-
Second Premolar	Right	0.306	<0.001**	0.278	<0.001**	<0.001**	<0.001**	-	-	-
	Left	0.781	<0.001**	0.154	<0.001**	0.110	0.011*	-	-	-
First Molar	Right	0.950	<0.001**	0.009**	<0.001**	0.042*	<0.001**	-	-	-
	Left	-	<0.001**	-	<0.001**	0.043*		-	-	-
Second Molar	Right	-	-	-	-	-	-	-	-	-
	Left	-	-	-	-	-	-	-	-	-

**Figure 1:**
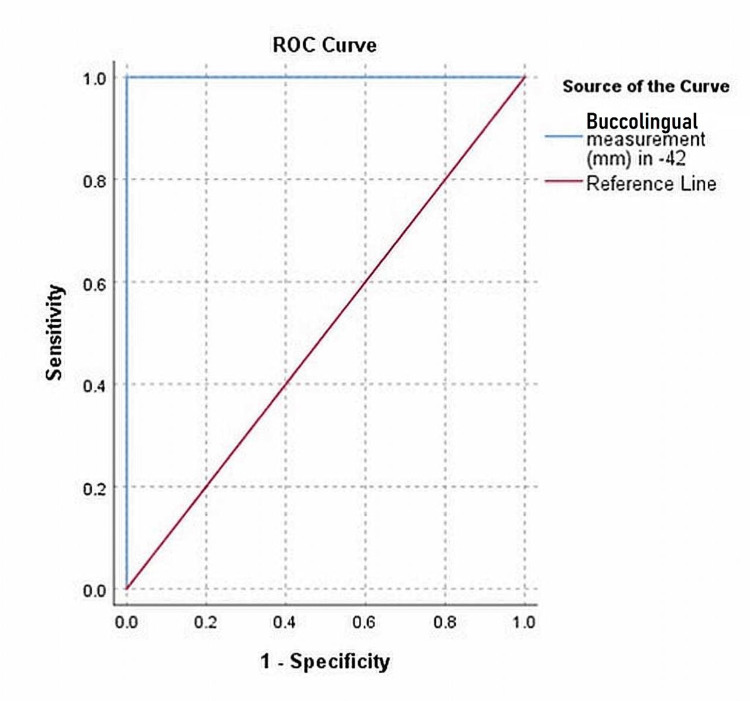
ROC analysis for the basic restorative need of mandibular right lateral incisor ROC: Receiver operating characteristic

The running ROC curve depicts the best cut-off value of the buccolingual measurement to predict none, preventive, basic restorative, advanced restorative, and rehabilitative TN for each tooth. The area under the curve (AUC) of ROC analysis represents the degree to which different TN can be distinguished at different threshold settings based on buccolingual measurements with the CAITN probe. However, the ROC analysis of the right mandibular central incisor for the advanced restorative need was laid under the reference diagonal line, reflecting the poor predictive performance of classifying the TN. The AUC takes into account both sensitivity and specificity, which are vividly shown in a single plot in Figure 1.

The maximum AUC of 1 in the ROC curve represents the perfect distinguishing ability to classify the TN based on the buccolingual dimension of the tooth, as shown in Tables 7-10.

**Table 7:** ROC of the maxillary arch (right quadrant) **: highly significant NPV: Negative predictive value; PPV: positive predictive value; AUC: area under the curve; ROC: receiver operating characteristic

Tooth	Treatment Needs	Cut-off value (mm)	Sensitivity (%)	Specificity (%)	PPV (%)	NPV (%)	Accuracy (%)	AUC	p-value
Second Molar	Preventive or more	≤ 9	100	91.67	75	100	93.33	0.958	<0.001**
	Basic restorative or more	-	-	-	-	-	-	-	-
	Advanced restorative or more	-	-	-	-	-	-	-	-
	Rehabilitative	-	-	-	-	-	-	-	-
First Molar	Preventive or more	-	-	-	-	-	-	-	-
	Basic restorative or more	≤ 10	100	100	100	100	100	1	<0.001**
	Advanced restorative or more	≤ 6	100	85.71	33.33	100	86.67	0.929	<0.001**
	Rehabilitative	-	-	-	-	-	-	-	-
Second Premolar	Preventive or more	≤ 8	100	100	100	100	100	1	<0.001**
	Basic restorative or more	≤ 8	100	80	90.91	100	93.33	0.940	<0.001**
	Advanced restorative or more	≤ 3	100	92.31	66.67	100	93.33	0.962	<0.001**
	Rehabilitative	-	-	-	-	-	-	-	-
First Premolar	Preventive or more	-	-	-	-	-	-	-	-
	Basic restorative or more	≤ 7	100	100	100	100	100	1	<0.001**
	Advanced restorative or more	≤ 3	100	92.31	66.67	100	93.33	0.962	<0.001**
	Rehabilitative	-	-	-	-	-	-	-	-
Canine	Preventive or more	≤ 6	100	100	100	100	100	1	<0.001**
	Basic restorative or more	≤ 6	100	88.89	85.71	100	93.33	0.972	<0.001**
	Advanced restorative or more	-	-	-	-	-	-	-	-
	Rehabilitative	-	-	-	-	-	-	-	-
Lateral Incisor	Preventive or more	≤ 5	100	100	100	100	100	1	<0.001**
	Basic restorative or more	≤ 4	100	100	100	100	100	1	<0.001**
	Advanced restorative or more	-	-	-	-	-	-	-	-
	Rehabilitative	-	-	-	-	-	-	-	-
Central Incisor	Preventive or more	≤ 6	100	100	100	100	100	1	<0.001**
	Basic restorative or more	≤ 6	100	90	83.33	100	93.33	0.980	<0.001**
	Advanced restorative or more	-	-	-	-	-	-	-	-
	Rehabilitative	-	-	-	-	-	-	-	-

**Table 8:** ROC of the maxillary arch (left quadrant) **highly significant NPV: Negative predictive value; PPV: positive predictive value; AUC: area under the curve ROC: receiver operating characteristic

Tooth	Treatment Needs	Cut-off value (mm)	Sensitivity (%)	Specificity (%)	PPV (%)	NPV (%)	Accuracy (%)	AUC	p-value
Second Molar	Preventive or More	-	-	-	-	-	-	-	-
	Basic Restorative or More	≤ 9	50	100	100	92.86	93.33	0.750	0.131
	Advanced Restorative or More	-	-	-	-	-	-	-	-
	Rehabilitative	-	-	-	-	-	-	-	-
First Molar	Preventive or More	≤ 11	100	100	100	100	100	1	<0.001**
	Basic Restorative or More	≤ 9	100	100	100	100	100	1	<0.001**
	Advanced Restorative or More	≤ 3	100	92.86	50	100	93.33	0.964	<0.001**
	Rehabilitative	-	-	-	-	-	-	-	-
Second Premolar	Preventive or More	-	-	-	-	-	-	-	-
	Basic Restorative or More	≤ 8	100	100	100	100	100	1	<0.001**
	Advanced Restorative or More	≤ 3	100	100	100	100	100	1	<0.001**
	Rehabilitative	-	-	-	-	-	-	-	-
First Premolar	Preventive or More	-	-	-	-	-	-	-	-
	Basic Restorative or More	≤ 8	100	100	100	100	100	1	<0.001**
	Advanced Restorative or More	-	-	-	-	-	-	-	-
	Rehabilitative	-	-	-	-	-	-	-	-
Canine	Preventive or More	-	-	-	-	-	-	-	-
	Basic Restorative or More	≤ 5	70	100	100	62.5	80	1	<0.001**
	Advanced Restorative or More	≤ 3	100	84.62	50	100	86.67	0.923	<0.001**
	Rehabilitative	≤ 3	100	78.57	25	100	80	0.821	<0.001**
Lateral Incisor	Preventive or More	≤ 6	100	100	100	100	100	1	<0.001**
	Basic Restorative or More	≤ 4	88.89	100	100	85.71	93.33	0.991	<0.001**
	Advanced Restorative or More	≤ 4	100	53.85	25	100	60	0.827	0.001**
	Rehabilitative	-	-	-	-	-	-	-	-
Central Incisor	Preventive or More	≤ 6	83.33	100	100	90	93.33	0.917	<0.001**
	Basic Restorative or More	≤ 6	100	100	100	100	100	1	<0.001**
	Advanced Restorative or More	≤ 3	100	100	100	100	100	1	<0.001**
	Rehabilitative	-	-	-	-	-	-	-	-

**Table 9:** ROC of the mandibular arch (left quadrant) **highly significant NPV: Negative predictive value; PPV: positive predictive value; AUC: area under the curve; ROC: receiver operating characteristic

Tooth	Treatment Needs	Cut-off value (mm)	Sensitivity (%)	Specificity (%)	PPV (%)	NPV (%)	Accuracy (%)	AUC	p-value
Second Molar	Preventive or More	-	-	-	-	-	-	-	-
	Basic Restorative or More	≤ 6	100	100	100	100	100	1	<0.001**
	Advanced Restorative or More	≤ 3	100	100	100	100	100	1	<0.001**
	Rehabilitative	-	-	-	-	-	-	-	-
First Molar	Preventive Or More	-	-	-	-	-	-	-	-
	Basic Restorative or More	≤ 9	100	100	100	100	100	1	<0.001**
	Advanced Restorative or More	≤ 5	100	92.31	66.67	100	93.33	0.923	<0.001**
	Rehabilitative	-	-	-	-	-	-	-	-
Second Premolar	Preventive or More	≤ 6	100	100	100	100	100	1	<0.001**
	Basic Restorative or More	≤ 5	100	100	100	100	100	1	<0.001**
	Advanced Restorative or More	≤ 2	75	90.91	75	90.91	86.67	0.818	<0.001**
	Rehabilitative	-	-	-	-	-	-	-	-
First Premolar	Preventive Or More	-	-	-	-	-	-	-	-
	Basic Restorative or More	≤ 7	100	100	100	100	100	1	<0.001**
	Advanced Restorative or More	≤ 6	100	50	33.33	100	60	0.819	<0.001**
	Rehabilitative	-	-	-	-	-	-	-	-
Canine	Preventive or More	≤ 7	100	100	100	100	100	1	<0.001**
	Basic Restorative or More	≤ 7	100	90	83.33	100	93.33	0.960	<0.001**
	Advanced Restorative or More	≤ 7	100	69.23	33.33	100	73.33	0.731	0.007**
	Rehabilitative	-	-	-	-	-	-	-	-
Lateral Incisor	Preventive or More	≤ 5	100	100	100	100	100	1	<0.001**
	Basic Restorative or More	≤ 4	100	100	100	100	100	1	<0.001**
	Advanced Restorative or More	≤ 4	100	78.57	25	100	80	0.821	0.001**
	Rehabilitative	-	-	-	-	-	-	-	-
Central Incisor	Preventive or More	≤ 5	100	100	100	100	100	1	<0.001**
	Basic Restorative or More	≤ 4	100	100	100	100	100	1	<0.001**
	Advanced Restorative or More	-	-	-	-	-	-	-	-
	Rehabilitative	-	-	-	-	-	-	-	-

**Table 10:** ROC of the mandibular arch (right quadrant) **: highly significant NPV: Negative predictive value; PPV: positive predictive value; AUC: area under the curve; ROC: receiver operating characteristic

Tooth	Treatment Needs	Cut-off value (mm)	Sensitivity (%)	Specificity (%)	PPV (%)	NPV (%)	Accuracy (%)	AUC	p-value
Second Molar	Preventive or More	-	-	-	-	-	-	-	-
	Basic Restorative or More	≤ 7	100	100	100	100	100	1	<0.001**
	Advanced Restorative or More	≤ 4	100	100	100	100	100	1	<0.001**
	Rehabilitative	-	-	-	-	-	-	-	-
First Molar	Preventive or More	≤ 7	77.78	100	100	75	86.67	0.870	<0.001**
	Basic Restorative or More	≤ 7	87.5	100	100	87.5	93.33	0.964	<0.001**
	Advanced Restorative or More	≤ 5	100	84.62	50	100	86.67	0.942	<0.001**
	Rehabilitative	-	-	-	-	-	-	-	-
Second Premolar	Preventive or More	≤ 6	88.89	100	100	85.71	93.33	0.963	<0.001**
	Basic Restorative or More	≤ 5	87.5	100	100	87.5	93.33	0.946	<0.001**
	Advanced Restorative or More	≤ 3	100	91.67	75	100	93.33	0.986	<0.001**
	Rehabilitative	-	-	-	-	-	-	-	-
First Premolar	Preventive or More	-	-	-	-	-	-	-	-
	Basic Restorative or More	≤ 4	100	100	100	100	100	1	<0.001**
	Advanced Restorative or More	≤ 5	100	66.67	42.86	100	73.33	0.861	<0.001**
	Rehabilitative	-	-	-	-	-	-	-	-
Canine	Preventive or More	-	-	-	-	-	-	-	-
	Basic Restorative or More	≤ 6	100	100	100	100	100	1	<0.001**
	Advanced Restorative or More	≤ 6	100	75	50	100	80	0.903	<0.001**
	Rehabilitative	-	-	-	-	-	-	-	-
Lateral Incisor	Preventive or More	-	-	-	-	-	-	-	-
	Basic Restorative or More	≤ 5	100	100	100	100	100	1	<0.001**
	Advanced Restorative or More	-	-	-	-	-	-	-	-
	Rehabilitative	-	-	-	-	-	-	-	-
Central Incisor	Preventive or More	≤ 5	80	100	80	90.91	93.33	0.900	<0.001**
	Basic Restorative or More	≤ 5	75	90.91	75	90.91	86.67	0.830	<0.001**
	Advanced Restorative or More	-	-	-	-	-	-	0.357	0.381
	Rehabilitative	-	-	-	-	-	-	-	-

A high sensitivity of the test corresponds to a high negative predictive value to "rule out" the classifier, and a higher specificity indicates a higher positive predictive value to "rule in" the test. The graphical plot displays the trade-off between true positive (sensitivity) and false positive (specificity) cases to determine the best cut-off value of the buccolingual dimension of the tooth.

## Discussion

The factors associated with cervical abrasion include overzealous tooth brushing using hard bristles and the use of abrasive toothpaste [14-17]. The tubules in sensitive dentin are said to be open between the exposed dentinal surface and the pulp and are wider than those in nonsensitive dentin. Furthermore, the number of tubules in the sensitive dentin is eightfold wider than the nonsensitive dentin [7]. It is stated that there is no ideal treatment for DH, even in the case of a combination of diverse protocols [18]. Two common methods to determine the intensity of DH are by asking some questions from the patient and the other is through clinical examination. The prevalence of DH through the questionnaire method is usually estimated as higher than that of the other method [7]. The primary goal of the study was to evaluate the depth of the cervical abrasions by measuring the buccolingual dimension of the tooth followed by determining the appropriate treatment of lesions. There is a variety of products and techniques available in the market to treat the condition [19] based on which the TN in this study was classified as none, preventive, basic restorative, advanced restorative, and rehabilitative TN.

The endodontist in our survey used myriad products to treat the abraded tooth. The results from this study aid clinicians and epidemiologists in identifying and deciding which teeth require restoration [20]. Although providing advice about diet or tooth-brushing was a common approach to the management of DH, most respondents did not consider it a successful clinical strategy [11]. We employed a one-second application that was calibrated by the clinician using an anemometer to measure the distance between the syringe tip and the anemometer vane face required to produce a 4 to 5 m/ second velocity. We chose the air velocity after conducting tests with a multitude of air syringes and tips to ensure that a reasonable space between the tip and the tooth surface (15-60 mm) was maintained [9]. The relationship between the buccolingual measurement of the abraded tooth and dentinal hypersensitivity was shown to be highly significant in the current investigation in all the teeth in the study sample except in the right and left mandibular central incisors. The mean buccolingual measurements of maxillary anterior teeth with respect to DH ranged from 3 mm to 3.67 mm on the right side and 2.75 mm to 4.5 mm on the left side; on the maxillary right premolars, it was 2.60 mm to 3.43mm and 4 to 4.29 mm on the left side; on the maxillary right molars, it was reported from 6 mm to 6.57 mm and 5.57 mm on the left maxillary molars. The mean buccolingual measurements of mandibular anterior teeth ranged from 3.5 mm to 5.5 mm on the right side and 3.33 mm to 5 mm on the left side; on the mandibular right premolars, it was 3.17 mm to 3.80 mm and 2.86 mm to 4.2 mm on the left side; on the maxillary right molars, it was reported from 3 mm to 5 mm and 3.5 mm to 5.17 mm on the left maxillary molars.

The buccolingual dimension of each tooth tends to decrease in size with severe abrasion. Accordingly, there was a statistically significant difference between the buccolingual dimension of the teeth and various TN categories. Contrary to the trend of decreasing buccolingual measurement of teeth from the no treatment to rehabilitative TN, an unusual increase in buccolingual measurement is observed in the left maxillary canine from 2 mm for advanced restorative TN to 3 mm for rehabilitative need and in the left mandibular canine (from 4.33 mm for the basic restorative need to 5.5 mm for advanced restorative need), right central (from 5 mm for the basic restorative need to 6 mm for advanced restorative need), and left lateral incisors (from 3 mm for the basic restorative need to 4 mm for advanced restorative need) in the mandibular arch. Such a variation in measurements could be attributed to a smaller sample size and individual threshold variations of DH.

These findings were further emphasized by the statistically insignificant difference observed in buccolingual measurements between the pair-wise comparisons of certain TN categories by applying the post hoc test. The buccolingual measurement between the no TN and preventive TN of the first molar, central, and lateral Incisors of the left maxillary arch was insignificant. Similarly, a statistically insignificant difference was noticed between the preventive and basic restorative TN in most of the teeth except in the left central incisor and right lateral incisor of the maxillary arch. The comparison of buccolingual measurement with the basic and advanced restorative TN was found to be significant for all the teeth with exception of the left lateral incisor and the right and left first molars of the maxillary arch. The rehabilitative TN based on the buccolingual measurements (3 mm) was estimated only for the maxillary left canine which was found to be insignificant for pair-wise comparison of basic restorative, advanced restorative, and rehabilitative needs.

Similarly, in the mandibular arch, an insignificant difference was observed between the comparison of none and preventive TN in the left lateral incisor, right first molar, and right and left second premolars. The pair-wise comparison of preventive and basic restorative TN based on buccolingual measurements was found to be significant only in the left central and lateral Incisors, and the right first molar in the mandibular arch. However, significant differences were revealed for all the maxillary and mandibular teeth for pair-wise comparison of none, basic and advanced restorative TN.

The best cut-off value of maxillary molars for preventive needs ranged from 9 mm to 11 mm; it was 9 to 10 mm for basic restorative needs and 3 mm to 6 mm for advanced restorative needs. Furthermore, the best cut-off value of maxillary premolars for basic restorative needs was 7 mm to 8 mm and for advanced restorative needs it was 3 mm. The best cut-off value of maxillary anterior teeth for preventive needs was 5 mm to 6 mm, for basic restorative needs it was 4 mm to 6 mm, and for advanced restorative needs it was 3 mm to 4 mm. Similarly, the best cut-off value of mandibular molars for preventive need was 7 mm and that of basic restorative TN ranged from 7 mm to 9mm and advanced restorative TN was from 3 mm to 5 mm. In premolars, the cut-off value ranged from 4 mm to 7 mm and 2 mm to 6 mm for basic and advanced restorative TN respectively. The best cut-off value of mandibular anterior teeth was from 4 mm to 7 mm for all the TN.

The summary measure of the ROC curve is the AUC which evaluates the accuracy of the predictive ability to discriminate the TN based on buccolingual measurements using the CAITN probe by determining the optimal cut-off values for each tooth. The lower cut-off values are more likely to identify patients with extreme loss of tooth structure due to severe cervical abrasion. The AUC of most of the maxillary and mandibular dentition in the study sample was 1 or close to 1 for all the TN, which infers that the model predicts the TN more precisely based on the buccolingual measurements. An AUC of 0.75 was observed for basic restorative need in the left maxillary second molar, and for advanced restorative need (0.731) in the left mandibular canine but was statistically highly significant. Nevertheless, an AUC of 0.357 (p>0.05) was reported for advanced restorative need in the right mandibular central incisor.

Lesions are more common in all age groups among people who brush their teeth horizontally and with abrasive materials, as shown by previous research studies [21]. Abrasive lesions exhibit significant variations with intensifying time and frequency of brushing, frequency of changing toothbrushes, and kind of toothpaste used [22,14]. The management of cervical abrasion necessitates a comprehension of the ambiguity of the lesion and also a thorough understanding of available therapeutic options. Differences in salivary flow and its composition may play a part in hypersensitivity advancement by negatively influencing the surface layer of the tooth or the deposition of intratubular dentin. Besides that, the biofilm of the gingiva, as well as profuse consumption of acidified beverages, invariably is accompanied by brushing the teeth shortly after consuming the beverage, which will eventually increase the susceptibility of the individual to DH [23].

There have been innumerable clinical trials on DH, with varying protocols. However, the clinical research literature as a whole is far off from the irrefutable suggestions of addressing one superior technique [24]. Realizing these concerns, the Canadian Advisory Board on Dentin Hypersensitivity in June 2002 recommended consensus-based guidelines on the clinical management of dentin hypersensitivity [25]. The novel CAITN probe could be utilized effectively as a beneficial tool to determine the depth of the lesion and its corresponding treatment needs [12].

Clinical implications and the scope of the study

The treatment of non-carious cervical lesions dictates identification of the problem, and diagnosis; thereby, removal of etiologic factors, monitoring, and treatment. A personalized therapeutic approach should be applied, with the appropriate strategy employed for the specific case scenarios. Moreover, the progression of the cervical abrasion is reportedly slow, but with a considerable variation among patients [18]. Henceforth, customized monitoring standards established in the present study using the CAITN probe assess the severity of the lesions, and its subsequent progression by determining the depth of the existing lesion. Additionally, the TNs suggested based on the depth of the lesion in the current study ensures a predictable and reliable treatment regimen for cervical abrasion with an emphasis on prevention, and treatment of dentin hypersensitivity.

The main limitation of diagnosing dentinal sensitivity is the subjective perception, which may differ for the same patient at different times. The participants enrolled were predominantly from urban and suburban settings. These findings might differ in other populations and may not reflect a truly random survey of the general population with cervical abrasion. There may be possible unknown sampling bias of selected study subjects since the sample essentially volunteered to be recruited into the present study. Furthermore, it is customary that there are substantial differences among endodontists in the treatment of cervical abrasion and individual variability in the threshold of dentinal sensitivity. The present study associated the buccolingual tooth dimension and depth of the cervical lesion to establish the appropriate TN. Nevertheless, we recognize that the decision on the optimal TN based on direct calibration of the depth of the cervical lesion will be within the scope of future research. Albeit with the given constraints, the present study enables a correct diagnosis of dental abrasions and allows determining the various TN with the most appropriate restorative material.

## Conclusions

The present study enables a correct diagnosis of cervical abrasions and determines the various TN with the most appropriate restorative material. The running ROC curve discloses the best cut-off value of the buccolingual measurement to predict the various categories of TN of each tooth. As the AUC was more close to 1 in the majority of the teeth in the sample, the model predicts the TN more precisely based on the buccolingual measurements. These baseline data help to design clinical studies that test relevant treatment and diagnostic strategies.
